# Adoption of Machine Learning in Intelligent Terrain Classification of Hyperspectral Remote Sensing Images

**DOI:** 10.1155/2020/8886932

**Published:** 2020-09-01

**Authors:** Yanyi Li, Jian Wang, Tong Gao, Qiwen Sun, Liguo Zhang, Mingxiu Tang

**Affiliations:** ^1^College of Geomatics, Shandong University of Science and Technology, Qingdao 266500, China; ^2^Chinese Society of Optimization, Overall Planning and Economic Mathematics, Beijing 100089, China; ^3^College of Ocean Science and Engineering, Shandong University of Science and Technology, Qingdao 266500, China; ^4^Shandong Provincial Institute of Land Surveying and Mapping, Jinan 250001, China

## Abstract

To overcome the difficulty of automating and intelligently classifying the ground features in remote-sensing hyperspectral images, machine learning methods are gradually introduced into the process of remote-sensing imaging. First, the PaviaU, Botswana, and Cuprite hyperspectral datasets are selected as research subjects in this study, and the objective is to process remote-sensing hyperspectral images via machine learning to realize the automatic and intelligent classification of features. Then, the basic principles of the support vector machine (SVM) and extreme learning machine (ELM) classification algorithms are introduced, and they are applied to the datasets. Next, by adjusting the parameter estimates using a restricted Boltzmann machine (RBM), a new terrain classification model of hyperspectral images that is based on a deep belief network (DBN) is constructed. Next, the SVM, ELM, and DBN classification algorithms for hyperspectral image terrain classification are analysed and compared in terms of accuracy and consistency. The results demonstrate that the average detection accuracies of ELM on the three datasets are 89.54%, 96.14%, and 96.28%, and the Kappa coefficient values are 0.832, 0.963, and 0.924; the average detection accuracies of SVM are 88.90%, 92.11%, and 91.68%, and the Kappa coefficient values are 0.768, 0.913, and 0.944; the average detection accuracies of the DBN classification model are 92.36%, 97.31%, and 98.84%, and the Kappa coefficient values are 0.883, 0.944, and 0.972. The results also demonstrate that the classification accuracy of the DBN algorithm exceeds those of the previous two methods because it fully utilizes the spatial and spectral information of hyperspectral remote-sensing images. In summary, the DBN algorithm that is proposed in this study has high application value in object classification for remote-sensing hyperspectral images.

## 1. Introduction

Remote-sensing hyperspectral technology is a comprehensive new technology. Remote-sensing hyperspectral images can effectively retain the spatial and spectral information of ground objects. Object detection has important application value in remote sensing, and the analysis of terrain changes can provide timely information regarding changes in large-scale ground objects on the Earth surface [1, 2]. Remote-sensing hyperspectral technology has been widely applied in agriculture, geology, and ecology [3]. Mastering the surface object information is of substantial significance for improving the surrounding environment. Therefore, the classification of remote-sensing hyperspectral images has important theoretical value and practical significance.

However, a hyperspectral image has high resolution and large data volume; hence, hyperspectral data should be detected using a more detailed method than those that are applied to traditional multispectral images. Traditional machine learning methods, such as SVM, are commonly used in the classification of hyperspectral remote-sensing images [4]. Traditional machine learning methods often require model training on a large amount of data, and the data that are used for the training must have similar distribution characteristics; hence, it is difficult to obtain training sample data for some machine learning methods [5, 6]. Deep learning is often applied in multiple fields. This method can be used in the recognition of objects, behaviours, and images, for example. Studies have shown that deep learning algorithms can be used in the feature extraction of remote-sensing image blocks [7]. However, relatively few applications of deep learning algorithms in the classification of hyperspectral remote-sensing images have been demonstrated.

## 2. Literature Review

### 2.1. Application of Machine Learning in Image Classification

Machine learning technology can learn through training data, then finds the development trend of data from the diversified database, and realizes the automatic processing of data analysis [8]. Machine learning has achieved excellent results in the processing of nonlinear data, such as image, text, and voice, while deep learning technology in machine learning has a stronger advantage in image recognition. Garcia-Floriano et al. proposed a method for classification and recognition of medical images that were based on support vector machines, and the results presented that the method could be effectively used in the diagnosis and classification of diseases [9]. Sudharshan et al. conducted a classification of breast tumor biopsy images based on deep learning and found that this method has high classification accuracy and does not require image labeling [10]. Now machine learning method is widely used in medical image recognition, but it is also studied in hyperspectral image processing. Li et al. constructed a classification model of hyperspectral images that was based on deep learning method to solve the shortcomings of traditional machine learning method [11]. Lv and Han proposed a method that was based on the multiple reduced kernel extreme learning machine, applied it to the efficient classification of hyperspectral images, verified it with PaviaU and other databases, and found that the model has a high classification effect [12]. Murphy and Maggioni proposed an unsupervised learning method for hyperspectral image clustering based on spatial regularized random walk, which was found to have lower computational complexity by marking [13]. At present, more experts and scholars have studied the application of machine learning and deep learning methods in hyperspectral image classification, but there is no precision assessment of object classification with different recognition algorithms.

### 2.2. Processing of Remote-Sensing Hyperspectral Image

Remote-sensing hyperspectral images have been widely used in military, medical, and agricultural monitoring fields. In the process of acquisition and transmission of hyperspectral images, they will be affected by illumination, atmosphere, and radiation; hence, there will be a lot of noise in hyperspectral images, which will affect the credibility of image data and bring inconvenience to subsequent processing and analysis [14, 15]. Therefore, much research is focused on the feature extraction of noise in hyperspectral images. Duan et al. proposed a multiscale total variation method, which was applied to the extraction of structural features in hyperspectral images, and the fusion of multiscale structural features insensitive to image noise was conducted by kernel principal component analysis. The results present that the method also has high robustness in the extraction of image structure features with intense noise [16]. Mishra et al. proposed a two-dimensional nonsecondary sampling wavelet transform method and applied it to the noise reduction of hyperspectral images. The results illustrate that even images with continuous noise of high wavelength can achieve automatic noise reduction [17]. Machine learning can remove the noise effectively in hyperspectral images, but the object detection in hyperspectral images has important significance for the application of remote-sensing technology. Zhou et al. proposed a method for hyperspectral image classification that is based on the compact and discriminative stacked autoencoder framework. After applying it to data classification, it is found that the method can effectively classify ground objects in hyperspectral images [18]. Hang et al. proposed a discrimination model, which is based on recurrent neural networks, and applied it to the discrimination of hyperspectral image learning features. The results illustrate that the model can extract spectral-spatial image features [19].

Machine learning algorithms can realize high classification accuracy in image classification and recognition, but relatively few studies have been conducted on the classification of ground objects in hyperspectral images. Therefore, a classification model that is based on SVM, ELM, DBN, and the spectrum-space characteristics of remote-sensing hyperspectral images is proposed. It is applied to three hyperspectral datasets, namely, PaviaU, Botswana, and Cuprite, and its accuracy is compared with those of various classification models in the terrain classification of hyperspectral image features. This study aims at providing a theoretical basis for increasing the efficiency of object recognition in remote-sensing hyperspectral images for realizing intelligent object recognition.

## 3. Methodology

### 3.1. Remote-Sensing Hyperspectral Image Segmentation Based on Spectral-Spatial Characteristics

**Figure 1:**
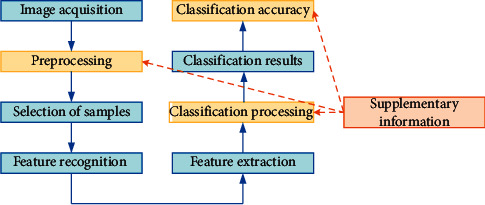
Classification process of remote-sensing hyperspectral images.

Different ground objects show different spectral characteristics and spatial distribution characteristics; hence, it is necessary to identify and judge image categories according to the information characteristics and spatial distribution characteristics of terrain spectral images [20]. Assuming that hyperspectral image data *ϖ*^*M*×*N*×*L*^ constitute a cube (where *M*, *N*, and *L* represent the length, width, and band, respectively, of the data), the classification performance of the data depends mainly on the image category, the dimension of the spectral data, the number of samples that are used during training, the classifier, and the classification method. Since the classification of hyperspectral data is similar to metaclassification, it can be followed from the whole variable space. A class of mean vectors is used to represent the coordinates in the eigenspace. The data are classified by using a classification function to divide the region. The classification process of hyperspectral images is illustrated in Figure 1.

As illustrated in Figure 1, the classification process of remote-sensing hyperspectral images can be divided into the following steps: (I) Image acquisition: the data from remote-sensing hyperspectral image databases being mainly used, namely, the University of Pavia (PaviaU) dataset, Botswana dataset, and Cuprite dataset; (II) image preprocessing, such as geometric calibration and atmospheric correction; (III) sample selection; (IV) recognition of features in an image; (V) feature extraction in image; (VI) classical disposal: in this study, SVM, ELM, and the deep learning algorithm being used to classify hyperspectral images; (VII) classification results; and (VIII) classification accuracy. The commonly used classification accuracy evaluation methods include overall classification accuracy, average classification accuracy, and Kappa value.

**Figure 2:**
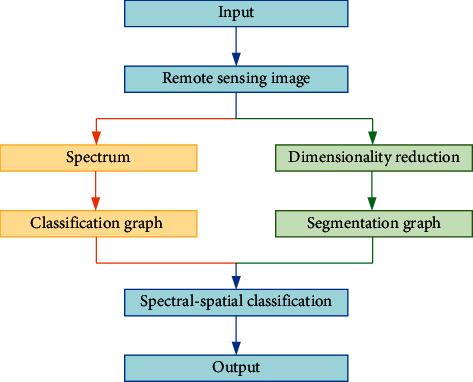
Spectral-spatial classification of remote-sensing hyperspectral images.

During the pretreatment of remote-sensing hyperspectral images, a method that is based on watershed and spatial regularization is mainly used to segment images. The spectral-spatial model classification framework is illustrated in Figure 2.

### 3.2. Brief Introduction to SVM and ELM

When using SVM to solve nonlinear problems, it is necessary to select a suitable kernel function and to map the samples in a low-dimensional space to a special space in a high-dimensional space. The optimal solution of the hyperplane is calculated in this space [21]. When nonlinear problem is solved by SVM, the expression of the nonlinear mapping is as follows:

(1) $$\begin{array}{c} x⟶\varphi \left(x\right). \end{array}$$

The above equation can be converted into the following equation:

(2) $$\begin{array}{c} Q\left(\alpha \right)=\sum_{i=1}^{n}\alpha_{i}+\frac{1}{2}\sum_{i,j=1}^{n}\alpha_{i}\alpha_{j}y_{i}y_{j}\varphi \left(x_{i}\right)\varphi \left(x_{j}\right). \end{array}$$

Among them, *φ*(*x*_*i*_)*φ*(*x*_*j*_) is the inner computing.

Nonlinear mapping can be used to solve nonlinear problems, but it increases the difficulty. Therefore, instead of kernel computation in a particular space, the space function *K* can be input:

(3) $$\begin{array}{c} K\left(x_{i},x_{j}\right)=\varphi \left(x_{i}\right)\varphi \left(x_{j}\right). \end{array}$$

Among them, *K*(*x*_*i*_, *x*_*j*_) is the kernel function.

Commonly used kernel functions include polynomial functions [(*x*_*i*_, *x*_*j*_)+*a*]^*q*^, Sigmoid function tanh[*v*(*x*_*i*_, *x*_*j*_)+*c*], redial basis kernel function exp(−(|*x* − *x*_*i*_|^2^)/*σ*^2^), B-spline kernel function *B*_2*N*+1_[*v*|*x* − *x*_*i*_|], and Fourier function ((sin(*N*+(1/2))(*x*_*i*_ − *x*_*j*_))/sin(1/2)(*x*_*i*_ − *x*_*j*_)).

In order to address the problem of generalization, penalty coefficient and relaxation factor are introduced to correct the SVM classification results.

ELM is widely used to solve various nonlinear problems due to its specific characteristics. Based on the ELM structure, ELM is mainly a feedforward neural network with a single hidden layer composed of an input layer, hidden layer, and output layer [22]. Assuming the random sample size is *N*, then the following equation can be obtained:

(4) $$\begin{array}{c} \left\{\begin{array}{c} X_{i}={\left[x_{i1},x_{i2},\ldots ,x_{\text{in}}\right]}^{T}\in R^{n},\\ t_{i}={\left[t_{i1},t_{i2},\ldots ,t_{\text{im}}\right]}^{T}\in R^{m}. \end{array}\right. \end{array}$$

Then, the expression of neural network with a single hidden layer is as follows:

(5) $$\begin{array}{c} o_{j}=\sum_{i=1}^{L}\beta_{i}g\left(W_{i}X_{j}+b_{i}\right),\;j=1,2,\ldots ,N. \end{array}$$

Among them, *g*(*x*) is an activation function; *β* is the output weight of each component of the hidden layer; *b*_*i*_ is the bias of each component of the *i*th hidden layer; and *W*_*i*_ is the input weight of each component of the *i*th hidden layer.

### 3.3. Image Classification Based on DBN

In the case of a great number of samples, unsupervised learning method gradually becomes an operational approach to machine learning. RBM is an unsupervised mapping learning method, which includes the input layer and hidden layer, and the connection between them is a full connection [23]. There is a connection weight between any two nodes in RBM. If the number of the hidden layer nodes in RBM is *N* and the number of input layer nodes is *M*, then the probability of activation of the hidden layer node *n*_*j*_ is as follows:

(6) $$\begin{array}{c} p\left(n_{j}\left|m\right.\right)=\sigma \left(b_{j}+\sum_{i}W_{i,j}m_{i}\right),\;i=1,2,\ldots ,M,j=1,2,\ldots ,N. \end{array}$$

Among them, *σ* is the activation function. Then, the probability of the hidden layer, the input layer, and node *m*_*i*_, which are activated, is as follows:

(7) $$\begin{array}{c} \left\{\begin{array}{c} p\left(n\left|m\right.\right)=\prod_{j=1}^{N}p\left(n_{j}\left|m\right.\right),\\ p\left(m_{i}\left|n\right.\right)=\sigma \left(c_{j}+\sum_{i}W_{i,j}n_{j}\right),\\ p\left(m\left|n\right.\right)=\prod_{i=1}^{M}p\left(m_{i}\left|n\right.\right). \end{array}\right. \end{array}$$

RBM training process is mainly divided into the following steps: (I) the data are input into the input layer, and the probability that the hidden layer and the input layer are activated is calculated by using equation (7). (II) After obtaining the distribution of each node in the hidden layer, Gibbs sampling method is used to extract the sample *n*_*j*_ in the hidden layer. (III) The sample *n*_*j*_ is used to reconstruct the input layer, and equation (7) is used to calculate the probability of the input layer being activated. (IV) After obtaining the different conditions of the reconstructed input layer nodes, Gibbs sampling method is used to extract *m*_*j*_ from the input layer samples. (V) After the reverse calculation, the activation probability and distribution probability of the hidden layer are obtained again. (VI) *w*+*λ*(*p*(*n*_*j*_|*m*_*i*_)*m*_*i*_ − *p*(*n*_*j*_′|*m*_*i*_′))⟶*W* is used to update the network weight, where *λ* represents the learning rate.

In this study, a single layer RBM that contains 50, 100, 150, 200, 250, and 300 hidden layer nodes is constructed, and the effects of the number of nodes on the spectral reconstruction performance and the classification accuracy are compared. Then, the number of unsupervised iterations is set as 50, 100, 200, 300, and 400 to evaluate the impact of the number of iterations on the classification accuracy. The learning rate in RBM is set as 0.01, 0.05, 0.1, 0.15, 0.3, and 0.45, and the performances at these learning rates are compared in terms of the classification accuracy.

**Figure 3:**
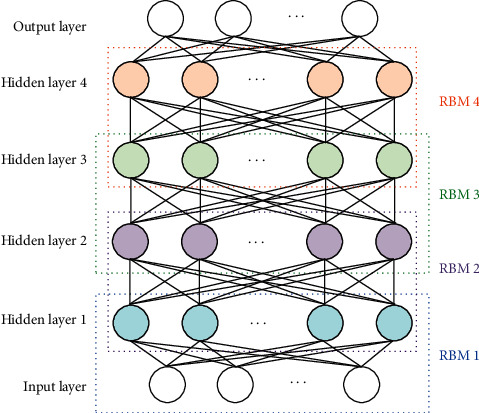
The basic structure of DBN.

The optimal RBM parameter is selected and DBN is built. DBN is composed of a multilayer RBM structure, and the training method of DBN is layer-by-layer training of RBM [24]. The basic structure of the DBN constructed based on RBM in this study is presented in Figure 3.

As illustrated in Figure 3, the classic DBN contains an input layer, a hidden layer, and an output layer. The structure contains four hidden layers and four RBM structures. In this study, the training methods for DBN are mainly divided into the following steps: (I) The data that must be trained are input into RBM1, and the training of DBN that is based on RBM is conducted using the RBM training method. (II) After the RBM training, the parameters of RBM1 are obtained, and RBM1 is used as the visible layer to train RBM2 via the same approach. (III) Similarly, all RBMs in DBN are obtained, and the initial parameter value of DBN is obtained after completion. Then, the network parameters are optimized. (IV) The contrastive wake-sleep algorithm is used to optimize and generate DBN, and the BP algorithm is used to optimize and discriminate DBN. (V) When the parameters are optimized by the BP algorithm, if the error between the actual value and the expected value of the output does not satisfy the requirements, backpropagation is conducted. The stochastic gradient descent method is used to correct the reverse parameters. When the number of iterations reaches the maximum and the target data have been obtained, the training is complete.

**Figure 4:**
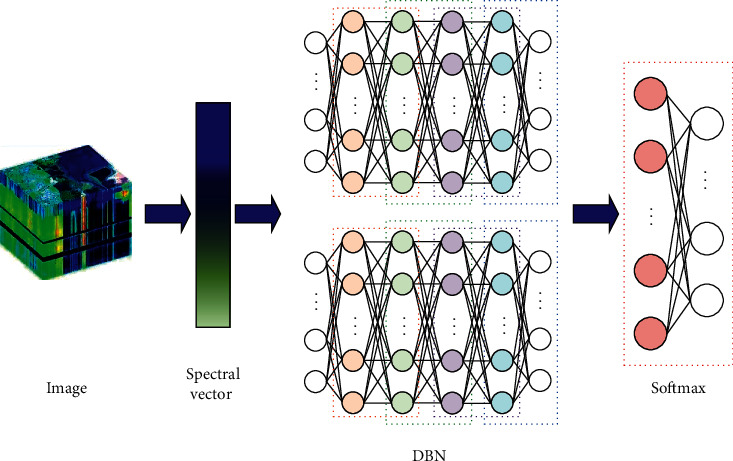
Classification framework of hyperspectral remote-sensing image based on DBN.

The basic framework of DBN-based terrain classification method for remote-sensing hyperspectral images, which is constructed in this study, is illustrated in Figure 4.

It is concluded from Figure 4 that the DBN-based terrain classification framework for remote-sensing hyperspectral images that is constructed in this study contains two layers of DBN, and the outermost layer of DBN is connected with a Softmax classifier. The Softmax classification layer optimizes the parameters in DBN via the BP method, and it can facilitate the direct output of the image category label.

## 4. Experiments and Results

The total number of samples that are used for model training in the PaviaU, Botswana, and Cuprite databases is 3000, and the number of samples for testing is 1000. When evaluating the model classification performance, the CPU is Intel i5−3470, dual-core, and 4GB of memory. ELM classification is realized in LibELM open interface. The SVM classification is C++ version. And DBN is MATLAB version. In order to better evaluate the effects of different methods on the classification of hyperspectral remote-sensing images, qualitative and quantitative evaluation methods are selected to evaluate the classification results. The quantitative evaluation indices include the time of classification use, the overall accuracy of classification, the average accuracy of classification, and Kappa coefficient.

### 4.1. Data Processing and Spectral Curve Analysis

**Figure 5:**
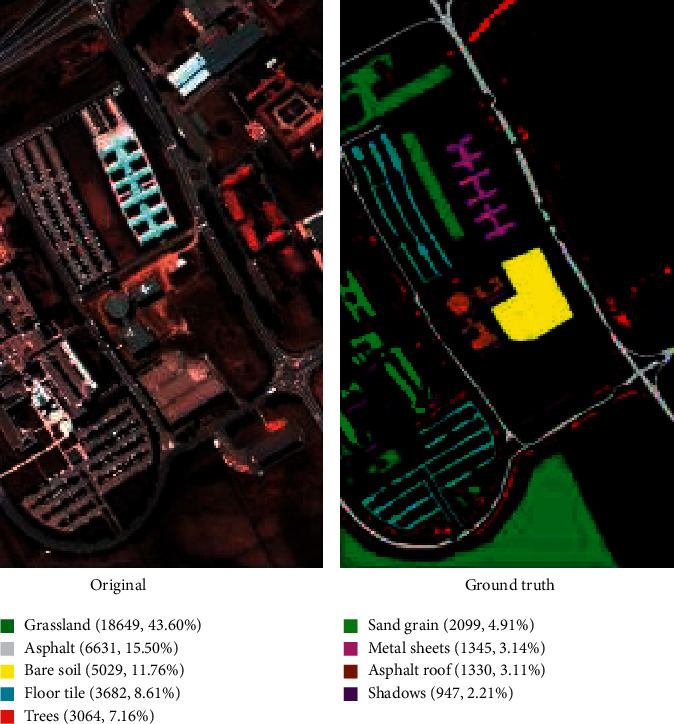
PaviaU data image and real ground objects map.

The PaviaU dataset is a remote-sensing hyperspectral image dataset that was collected by the university of Pavia in Italy in 2002 based on ROSIS sensor, which contains 115 spectral bands with a wavelength range of 0.43 ∼ 0.86 *μ*m. The size of the dataset is 610 ^*∗*^ 340 pixels, and according to Figure 5, the image data contain mainly information on 9 land types: asphalt road (15.50%), grassland (43.60%), sand grain (4.91%), trees (7.16%), sheet metal (3.14%), bare soil (11.76%), asphalt roof (3.11%), floor tile (8.61%), and shadow (2.21%). The spatial resolution of the information is approximately 1.3 m.

**Figure 6:**
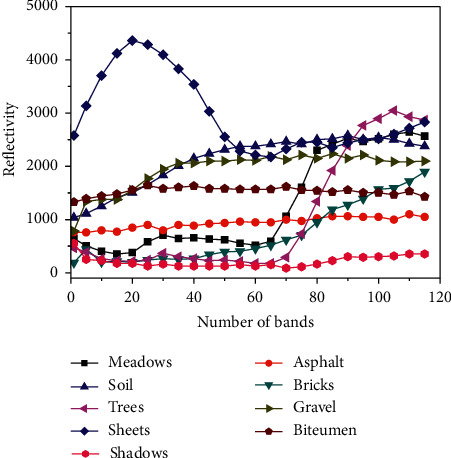
The spectral curve of each category of ground object in the PaviaU data image.

The spectral characteristics of ground objects are compared, and the reflectance is output once for every 5 bands. According to Figure 6, metal sheets, trees, grassland, and sand grain in the remote-sensing image data set of PaviaU show large differences in the reflectance spectra of ground objects in the visible and near-infrared bands. The reflectance patterns of the bare soil and sand grain categories are highly similar. Only a small difference is observed in the red-near-infrared band.

**Figure 7:**
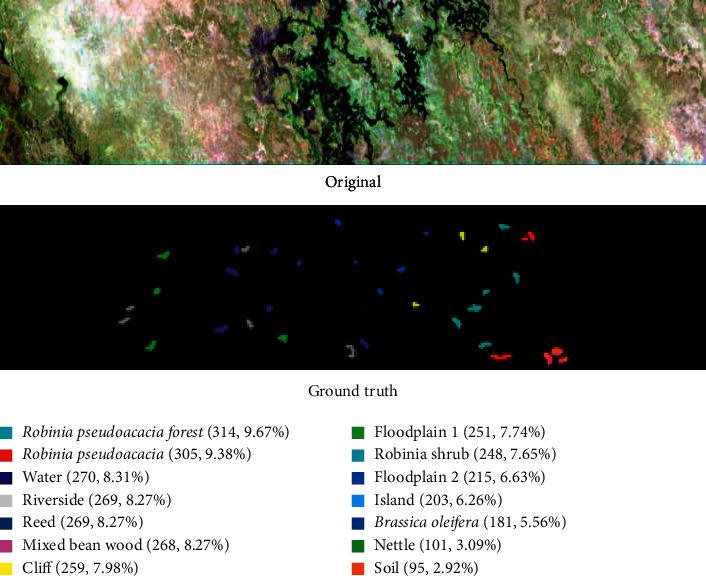
Botswana data images and the real ground object map.

The concentration dataset consists of image data of the Botswana delta that were collected in 2001 using a Hyperion EO-1 sensor, which senses 145 spectral bands with a wavelength range of 0.4 ∼ 2.5 *μ*m. The size of the dataset is 1476 ^*∗*^ 256 pixels.  Figure 7 shows that the dataset consists mainly of 14 types of terrain information: water (8.31%), nettle grass (3.09%), flood plain grassland 1(7.74%), flood plain grassland 2(6.63%), reed (8.27%), riverside (8.27%), cliff (7.98%), island (6.26%), *Robinia pseudoacacia* forest (9.67%), *Robinia* shrub (7.65%), *Robinia pseudoacacia* (9.38%), *Brassica oleifera* (5.56%), mixed bean wood (8.27%), and bare soil (2.92%). In addition, the spatial resolution of the information is 30 m.

**Figure 8:**
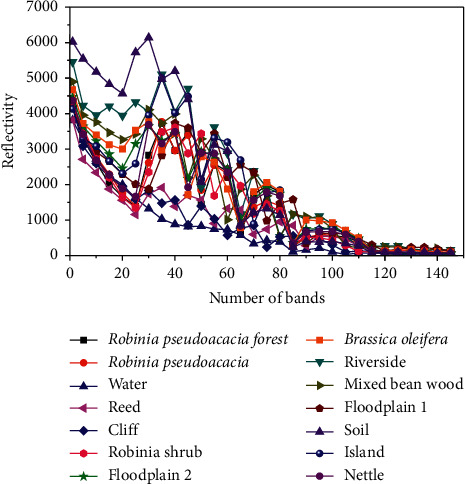
The spectral curve of each category of ground objects in the Botswana data image.

The spectral characteristics of features are compared, and the reflectance is output once every 5 bands. According to Figure 8, features of ground objects such as water, nettle grass, and bare soil in the Botswana remote-sensing image data vary substantially in the visible-light shortwave infrared region, while the spectral curves of *Robinia pseudoacacia* forest, Robinia shrub, and *Robinia pseudoacacia* are not easily distinguished nor are the ground object categories, such as *Brassica oleifera* and *Robinia* shrub.

**Figure 9:**
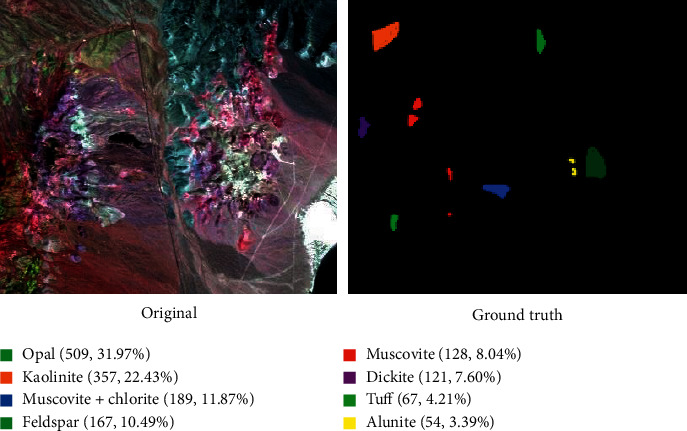
Cuprite data images and the real ground object map.

The Cuprite dataset consists of AVIRIS hyperspectral image data that were obtained by the United States Geological Survey in 1995. There are 50 spectral bands in the wavelength range of 1.99 ∼ 2.48 *μ*m in the image data. The size of the dataset is 350 ^*∗*^ 400 pixels. As shown in Figure 9, there are 8 main types of land information in this dataset: muscovite (8.04%), muscovite + chlorite (11.87%), tuff (4.21%), opal (31.97%), dickite (7.60%), kaolinite (22.43%), alunite (3.39%), feldspar (10.49%), muscovite (8.04%), muscovite + chlorite (11.87%), tuff (4.21%), opal (31.97%), dickite (7.60%), kaolinite (22.43%), alunite (3.39%), and feldspar (10.49%). The space rate of the information is approximately 20 m.

**Figure 10:**
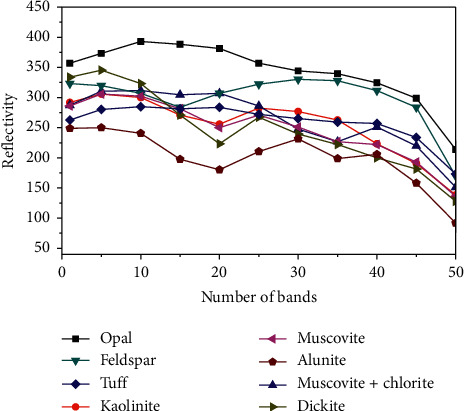
The spectral curve of each category of ground objects in the Cuprite data image.

The spectral characteristics of features are compared, and the reflectance is output once for every 5 bands. According to Figure 10, the spectral characteristics of ground objects such as opal and alunite in the dataset differ significantly in the range of the shortwave infrared region, whereas the spectral characteristics of kaolinite, tuff, and other ground objects are highly similar.

### 4.2. Influence of the Parameter Settings on the Classification Accuracy of the DBN Model

**Figure 11:**
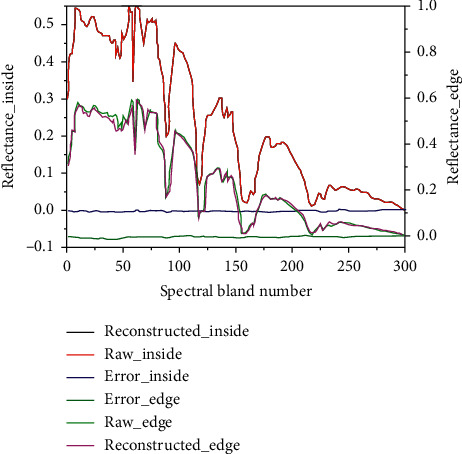
Image spectral reconstruction based on DBN model.

In this study, the constructed DBN model is used for spectral reconstruction of interior and boundary points of remote-sensing images. When the number of hidden layer nodes in DBN model is 200, the image in the PaviaU database is identified. According to Figure 11, the spectral reconstruction performance of DBN model on interior points of ground objects is higher than that on boundary points of ground objects. Therefore, the interior feature points of ground objects in remote-sensing hyperspectral images are selected for the final experiments.

**Figure 12:**
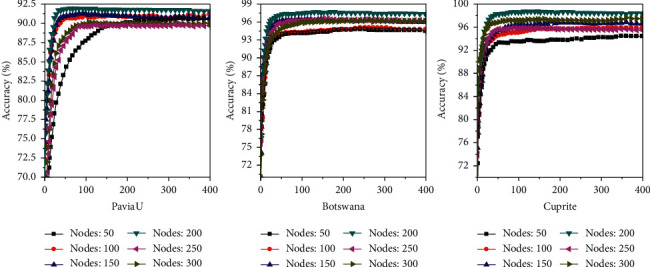
The influence of the number of hidden layer nodes on the recognition accuracy.

The learning rate is set at 0.01, and the number of unsupervised training iterations is 400. The effects of the number of nodes in hidden layers (50, 100, 150, 200, 250, and 300) in the PaviaU, Botswana, and Cuprite databases on the accuracy of DBN model recognition are compared. It is concluded from Figure 12 that when the number of the hidden layer nodes in the network is 200, the recognition accuracy is the highest. The recognition accuracies for images in the PaviaU, Botswana, and Cuprite databases are 91.93%, 97.59%, and 98.73%, respectively. Therefore, in this study, the number of the hidden layer nodes in the DBN model is set to 200 for subsequent experiments.

**Figure 13:**
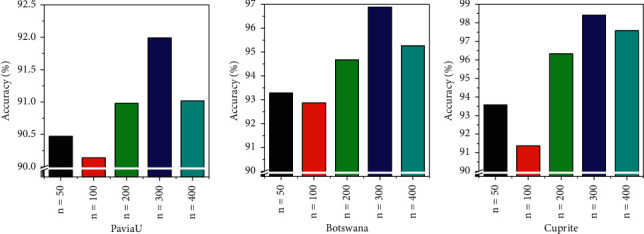
The influence of unsupervised training on recognition accuracy.

The effect of the number of unsupervised training iterations on the accuracy of DBN model recognition is evaluated. As shown in Figure 13, when the number of unsupervised training iterations is 100, the recognition accuracies of images in the PaviaU, Botswana, and Cuprite databases are the lowest, namely, 90.14%, 92.87%, and 91.37%, respectively. When the number of unsupervised training iterations is 300, the recognition accuracies of images in the PaviaU, Botswana, and Cuprite databases are the highest, namely, 91.99%, 96.88%, and 98.41%, respectively. Therefore, in this study, the number of unsupervised training iterations of the DBN model is set at 300 for subsequent experiments.

**Figure 14:**
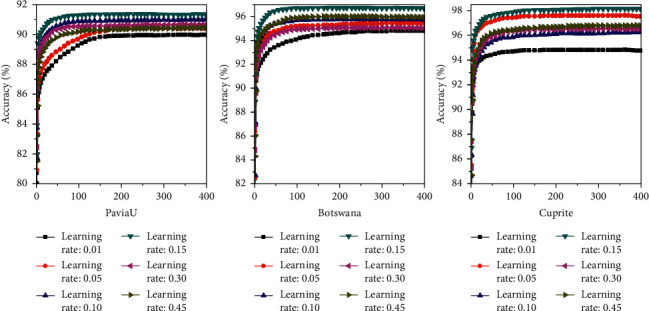
The influence of learning rate on recognition accuracy.

Then, the influence of the learning rate on the recognition accuracy of the DBN model is evaluated. As presented in Figure 14, when the learning rate is 0.01, the recognition accuracies of images in the PaviaU, Botswana, and Cuprite databases are the lowest, namely, 89.97%, 94.80%, and 94.75%, respectively. When the learning rate is 0.15, the recognition accuracies of images in the PaviaU, Botswana, and Cuprite databases are the highest, namely, 91.33%, 96.69%, and 98.15%, respectively. Therefore, the learning rate of the DBN model is set as 0.15 for subsequent experiments.

### 4.3. Comparison of Image Classification Results Based on the Three Classification Algorithms

**Figure 15:**
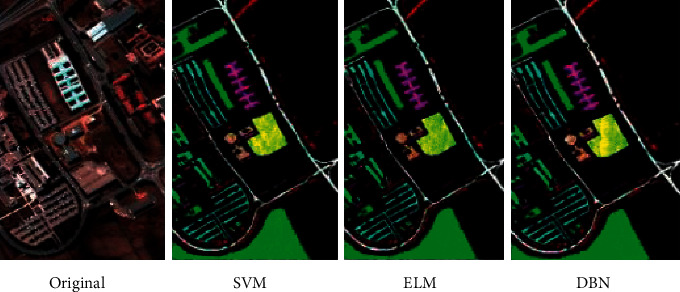
The classification results of PaviaU data.

The results of SVM, ELM, and DBN in the classification of PaviaU images are evaluated.  Table 1 illustrates that the classification time of ELM model is the shortest (32.17 s) while the classification time of SVM is the longest (605.36 s). The DBN model has the highest overall classification accuracy and average classification accuracy (90.54%, 92.36%) while the SVM model has the lowest overall classification accuracy and average classification accuracy (86.17%, 88.90%). The Kappa coefficients of ELM, SVM, and DBN are 0.832, 0.768, and 0.883, respectively. It can be concluded from Figure 15 that SVM, ELM, and DBN can effectively complete the classification of ground objects in PaviaU data images, but DBN has higher classification accuracy.

**Figure 16:**
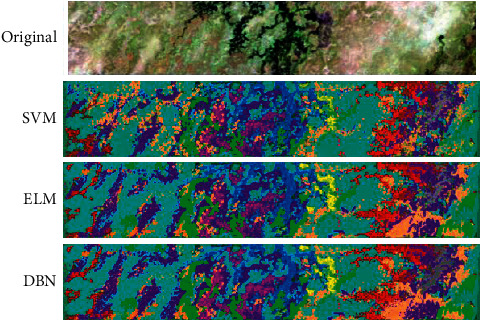
The classification results of Botswana data.

The effect of ELM, SVM, and DBN on the feature classification in the Botswana data image is compared. It can be found from Table 2 that the classification time of ELM is the shortest (34.55 s) and the SVM classification time is the longest (330.91 s). After comparing the classification accuracy, the overall classification accuracy and average classification accuracy of DBN are the highest (98.17%, 97.31%). The Kappa coefficients of ELM, SVM, and DBN models are 0.963, 0.913, and 0.944, respectively. As shown in Figure 16, the accuracy of DBN model and ELM model in the classification of image features is significantly higher than that of SVM model.

**Figure 17:**
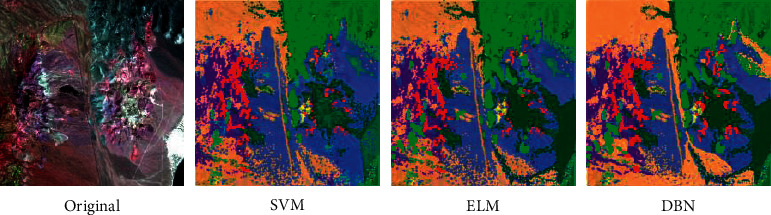
The classification results of Cuprite data.

The effect of ELM, SVM, and DBN on the terrain classification in Cuprite data image is compared. It can be concluded from Table 3 that the classification time of ELM is the shortest (24.83 s) and the classification time of SVM is the longest (35.22 s). After comparing the classification accuracy, the overall classification accuracy and average classification accuracy of DBN are the highest (99.06%, 98.84%). The Kappa coefficients of ELM, SVM, and DBN models are 0.924, 0.944, and 0.972, respectively. As shown in Figure 17, the classification accuracy of image features of DBN model is obviously better than that of SVM model and ELM model.

## 5. Discussion

The spectral feature of a ground object is its electromagnetic radiation, which includes reflection, and the band characteristic is determined by measuring the visible or invisible light absorption. Ground objects differ in terms of reflectivity, and reflectivity is often used for analysis. After analysing the spectral characteristics of objects in each dataset, it is found that the spectral morphologies of bare soil and sand grains in the PaviaU dataset are highly similar, and only a small difference is observed in the red-near-infrared band. Ground object categories such as *Robinia pseudoacacia* forest, *Robinia* shrub, and *Robinia pseudoacacia* in the Botswana dataset are affected by factors such as mixed pixels; hence, the spectral curves of these ground object categories are difficult to distinguish [25]. In addition, plants such as *Brassica oleifera* and *Robinia* shrubs also exhibit symbiosis in the concentrations of the Botswana data, which can lead to similar spectral curves of these ground objects. The spectral characteristics of ground object categories such as Kaolinite and Tuff in the Cuprite dataset are highly similar [26]. Analysis of the spectral characteristics of various types of objects is of substantial significance for increasing the classification accuracy and evaluating the classification performance of a ground object classification model.

DBN is a probabilistic generation model, and it is composed of multiple RBM layers. The DBN model is widely used in image recognition, and it has produced excellent results. Based on the DBN framework, Samadi et al. proposed a method for change detection in SAR images, and it was demonstrated that the method realizes high accuracy and detection performance [27]. Ahmad et al. proposed an algorithm for the automatic segmentation of liver CT image features that is based on DBN, and they found that the accuracy of this method was up to 94.80% [28]. Therefore, in this study, DBN is used to evaluate the spectral reconstruction of interior and boundary points of terrain images. The results demonstrate that the errors of spectral reconstruction of terrain images based on interior points are significantly lower than those based on boundary points; hence, in terrain classification, the spectral reconstruction performance of the classification network that selects the internal equinox of the image is higher than that of the network that selects the boundary points, which may be why there is much spectral confusion at the boundary points [29]. The classification time of a model is affected by many factors, such as the tools that are used in the calculation, the complexity of the model, and the quality of the data [30]. In this study, it is found that when ELM, SVM, and DBN models are used for hyperspectral image classification, the ELM model has the shortest classification time. However, the classification time of the DBN model that is proposed in this study is between those of the ELM model and the SVM model. This is because the DBN model that is constructed in this study contains 4 layers of RBMs; hence, the complexity of this model is high [31]. Subsequently, the Kappa coefficient is used to compare the accuracies of classification and identification of the models. The closer the Kappa coefficient is to 1, the higher the consistency of classification [32]. In this study, it is found that the Kappa coefficients of the DBN-based hyperspectral image feature classification model in PaviaU, Botswana, and Cuprite database image recognition are 0.883, 0.944, and 0.972, respectively, and the Kappa coefficients all exceed 0.75; hence, the classification model of hyperspectral image features that is based on DBN has high classification accuracy. This is consistent with the research results of Li et al. [33]. In addition, the Kappa coefficients of the SVM and ELM models exceed 0.75; thus, these two methods can also effectively classify ground objects, but their classification accuracies are lower than that of DBN. Therefore, DBN has higher robustness for spectral feature recognition and classification in hyperspectral images, which is consistent with the findings of Maggu et al. that the image classification model that is based on DBN has high robustness [34]. Previous studies on the classification and recognition of remote-sensing hyperspectral images focus mainly on the spectral dimension characteristics of image elements [35]. However, due to the complexity and the presence of mixed pixels in natural images, it is not sufficient to analyse the spectral characteristics of pixels. Therefore, the spectral characteristics and spatial characteristics of ground objects are analysed in the study. The study aims at increasing the classification accuracies of various types of ground objects in remote-sensing hyperspectral images. Understanding the natural variations of ground objects is of substantial significance. In the future, machine learning algorithms can be further investigated from various aspects, such as their loss function curves, to increase the accuracy and performance in ground object classification of remote-sensing hyperspectral images.

## 6. Conclusions

To study the performance of machine learning on terrain recognition and classification of remote-sensing hyperspectral images, an image classification model that is based on DBN is constructed. It is applied to the classification of real hyperspectral image data, and its classification performance is compared with those of SVM and ELM models. The results are as follows:

- Spectral curves that differ in terms of the types of ground object information have higher similarity, which increases the difficulty of classification of large datasets and affects the accuracy of classification of different types of ground objects.
- Based on the spectral characteristics and spatial characteristics of ground objects, the ground objects in remote-sensing hyperspectral images are classified, which lays a foundation for increasing the classification accuracies of various algorithms.
- The DBN model that is constructed in this study can effectively extract features from hyperspectral images and classify various types of ground objects.
- The DBN model that is constructed in this study outperforms the SVM and ELM models in terms of classification performance in the classification of ground objects in remote-sensing hyperspectral images.

However, strong spatial dimensional texture information and more noise are present in hyperspectral images, and the impacts of these factors on the classification performance have not been considered. Therefore, it is necessary to combine filtering and texture enhancement to increase the classification accuracy of the model. The results of this study can provide a theoretical basis for increasing the efficiency of terrain classification in remote-sensing hyperspectral images.

## Figures and Tables

**Table 1:** The results of classification evaluation indices of PaviaU data.

Indices	ELM	SVM	DBN
Classification time (s)	32.17	605.36	336.19
Overall classification accuracy (%)	86.32	86.17	90.54
Average classification accuracy (%)	89.54	88.90	92.36
Kappa coefficient	0.832	0.768	0.883

**Table 2:** The results of classification and evaluation indices of Botswana data.

Indices	ELM	SVM	DBN
Classification time (s)	34.55	330.91	127.64
Overall classification accuracy (%)	96.39	93.26	98.17
Average classification accuracy (%)	96.14	92.11	97.31
Kappa coefficient	0.963	0.913	0.944

**Table 3:** The results of classification and evaluation indices of Cuprite data.

Indices	ELM	SVM	DBN
Classification time (s)	24.83	35.22	25.39
Overall classification accuracy (%)	97.74	95.27	99.06
Average classification accuracy (%)	96.28	91.68	98.84
Kappa coefficient	0.924	0.944	0.972

## Data Availability

The data used to support the findings of this study are available from the corresponding author upon request.
